# Small Incision Lenticule Extraction for Postkeratoplasty Myopia and Astigmatism

**DOI:** 10.1155/2016/3686380

**Published:** 2016-06-30

**Authors:** Tamer H. Massoud, Osama Ibrahim, Kitty Shehata, Moones F. Abdalla

**Affiliations:** ^1^Ophthalmology Department, Alexandria University, Alexandria 21511, Egypt; ^2^Roayah Laser Vision Correction Centre, Alexandria, Egypt

## Abstract

*Purpose*. To evaluate the visual and refractive outcomes after small incision lenticule extraction (SMILE) for treating myopia and myopic astigmatism after penetrating keratoplasty (PKP).* Design*. Case-series.* Methods*. Ten eyes of 10 patients with previous PKP and residual myopic astigmatism for whom pentacam imaging and thickness measurements were acceptable for laser vision correction. Manifest refraction (MR), uncorrected distance visual acuity (UDVA), and corrected distance visual acuity (CDVA) were obtained preoperatively and one day, one week, and one, 3, and 6 months postoperatively. Cases were operated on the VisuMax® femtosecond laser platform with 500 kHz repetition rate.* Results*. The mean correction ratio for spherical errors was 0.84 ± 0.19 D and for the mean refractive spherical equivalent (MRSE) was 0.79 ± 0.13 D. Vector analysis showed a mean astigmatism reduction at the intended axis of 67 ± 25.25%, a correction index of 0.81 ± 0.21, and an overall mean percentage of success of astigmatism surgery of 53 ± 37.9%. The postoperative MRSE was stable throughout the 6-month follow-up period. The efficacy index was 0.93 and the safety index was 1.12.* Conclusion*. SMILE for correction of post-PKP myopia and astigmatism is effective, safe, and stable with moderate accuracy and predictability. Centration of the treatment within the grafts was easily performed.

## 1. Introduction

Achieving emmetropia in eyes with full thickness corneal grafts is a target that has long been pursued. For decades, a successful keratoplasty was judged in terms of preservation of a clear surviving graft as a final outcome. However, the ultimate goal of vision restoration was often hampered by the frequent association with postoperative ametropia and/or anisometropia [1, 2]. Astigmatism, the main refractive error following penetrating keratoplasty (PKP), has been related to a variety of pre-, intra-, and postoperative factors, while the less commonly associated spherical errors as myopia and, rarely, hyperopia were attributed mainly to axial length abnormalities and postoperative suture manipulations [1–4].

A wide scope of therapeutic modalities has been proposed and employed for the correction of such refractive errors. These ranged from the very conservative spectacle prescription to the final possibility of repeating the whole grafting procedure. However, none of these techniques has proven itself as a sole ideal solution for the management of post-PKP ametropia and many are associated with graft survival and, even, vision threatening complications [1, 2, 5].

Visual rehabilitation using spectacles represents a good choice but their use is limited by significant anisometropia especially with astigmatism more than 3 D or the presence of irregular astigmatism. Contact lenses (especially rigid gas permeable) provide another conservative management option but dry-eye syndrome and fitting-related inconvenience as well as patient's age, dexterity, and lifestyle are major concerns that may affect contact lens tolerance. They may also induce peripheral corneal neovascularization which can result in graft rejection [1, 2, 6–9].

Initial surgical management options range from simple procedures like selective suture removal [4] or suture adjustment [4, 10, 11] to the more sophisticated techniques of astigmatic keratotomy [12], relaxing incisions with or without compression sutures [13–15], and wedge resection [16]. The latter two have low predictability and are associated with a high incidence of recurrence of astigmatism. They also carry the risk of inducing overcorrection, perforation, wound dehiscence, and graft rejection. Moreover, they mainly aim for rectifying the astigmatic component of the refractive error but they do little, if any, when it comes to the spherical part of refraction [1, 2].

Intracorneal ring segment (ICR) implantation has recently been reported in post-PKP eyes but with significant undercorrection and low predictability compared to their results in keratoconic eyes. Immune reaction induction leading to graft rejection and ring migration and perforation into the anterior chamber are possible complications that can violate the future integrity of the grafted cornea [1, 17, 18].

Excimer laser photorefractive keratectomy (PRK) has also been proposed but was proven less predictable than when performed in previously nonoperated eyes. Limited astigmatic correction, irregular astigmatism induction, significant regression, corneal haze, and photoablation induced graft rejection are other important disadvantages of such a technique [19, 20]. The use of Laser in situ Keratomileusis (LASIK) reduced haze and allowed for more refractive correction but showed an increased risk of flap complications compared to normal eyes. Moreover, the lamellar keratectomy step causes thinning of the graft-host interface as the flap usually has a larger diameter than the corneal button. This disruption of the healing scar can add to the risk of having wound dehiscence. Also, flap adhesion is markedly reduced in eyes with low endothelial cell count. Corneal decompensation and graft failure were reported in such cases [20–26].

Toric intraocular lens implantation (phakic or pseudophakic) provides a wider range of correction but lens rotation, increased endothelial cell loss, corneal decompensation, graft rejection, endophthalmitis, and secondary glaucoma are important drawbacks of such procedures [27–32].

Small incision lenticule extraction (SMILE) is a novel, all-in-one, corneal laser refractive surgical procedure in which a lenticule of stromal tissue of planned thickness and diameter is isolated between two intracorneal planes created using a femtosecond laser platform. The lenticule is, then, manually removed from the cornea through a small incision to change the corneal curvature and exert its refractive effect. It was reported to be safe, predictable, and effective for treating myopia and myopic astigmatism in previously nonoperated eyes. It has the advantages of being flapless and less invasive than other intraocular procedures together with having the ability to tailor and center the whole procedure as required within a specific area of the cornea [33–35].

This study was performed with the aim of evaluation of visual and refractive outcomes after SMILE for treating post-PKP myopia and myopic astigmatism.

## 2. Patients and Methods

### 2.1. Patients

This interventional case-series included ten eyes of 10 patients with previous PKP and residual compound myopic astigmatism.

Inclusion criteria consisted of patients who had had an 8.25 mm donor button transplanted to 8.00 mm trephination of the recipient cornea with a duration of at least 18 months since the time of keratoplasty and a residual myopic refractive error of up to −10.0 diopters (D) of spherical equivalent with astigmatism up to 6.0 D at the spectacle plane. A smooth postkeratoplasty course with no attacks of graft rejection or suture complications was mandatory. Sutures were completely removed prior to performing the preoperative examination by at least three months during which patients were followed up monthly to ensure a stable refraction. Only patients whose topography and anterior and posterior elevation maps' data within the acceptable range for laser vision correction and a thinnest graft location of 500 *μ*m or greater were enrolled in the study.

Patients with graft apposition abnormalities (override or underride), severe dry eye, ocular surface disease, abnormal topographies, thin grafts, elevated intraocular pressure (IOP), peripheral corneal neovascularization, nonsuture track related peripheral opacities, or central or paracentral opacities were excluded. Patients experiencing post-SMILE interface inflammation, cellular infiltration, or any other reported SMILE complication were planned to be excluded, as well. Also patients with other ocular or recorded eye-related systemic illnesses (e.g., diabetes mellitus) were not included in the study.

The study was conducted between 2011 and 2015. The study protocol was based on the tenets of the Declaration of Helsinki and was approved by the ethics committee of the Faculty of Medicine of Alexandria University. The risks and advantages of the procedure were explained to all patients and an informed consent was obtained from each of them.

### 2.2. Preoperative and Postoperative Examination

Preoperatively, all patients had their detailed ocular and medical history taken. Full ophthalmic examination was performed including measurements of manifest refraction (MR), uncorrected distance visual acuity (UDVA), corrected distance visual acuity (CDVA) (using Snellen Decimal notation), and intraocular pressure as well as biomicroscopic fundus examination. Keratometric data, corneal topography, thickness data, and height maps of the anterior and posterior corneal surfaces were obtained from the Allegro Oculyzer*™* Pentacam (WaveLight, GmbH, Germany).

Following SMILE, postoperative follow-up visits were scheduled at 1 day, 1 week, and 1, 3, and 6 months postoperatively. During these visits, biomicroscopic examination of the anterior and posterior segments and UDVA, MR, and CDVA testing and recording were performed. Efficacy was expressed in terms of the cumulative UDVA at 6 months postoperatively as well as the efficacy index calculated as the ratio of the postoperative UDVA to the preoperative CDVA. Safety was judged by the change in the corrected distance visual acuity at 6 months postoperatively and also by the safety index calculated as the ratio of the postoperative CDVA to the preoperative CDVA.

### 2.3. Surgery

Cases were operated by two surgeons (Osama Ibrahim and Tamer H. Massoud) in Roayah Vision Correction Centre, Alexandria, Egypt. Preoperatively, refractive data was fed and revised on the computer system linked to the VisuMax femtosecond laser system (Carl Zeiss Meditec AG, Germany) with a 500 kHz repetition rate. Data entered included the MR to be corrected (measured at 12 mm vertex distance), the mean corneal radius (mm), or mean *k*-reading (D) in addition to pachymetry of the thinnest corneal (graft) location (obtained from Pentacam).

For all cases, small suction cups were chosen as the patient-laser interface. The cap and the lenticule diameters were calculated to be smaller than those of the graft (8.0 mm) so that they are centered within its margins. Corneal caps were planned to be 100 *μ*m thick for all cases. Their diameters ranged from 6.9 to 7.5 mm depending on the clear area of the graft available for refractive correction. The incision was placed at 120° to fit both right-handed surgeons. The width of the incision ranged from 3.4 to 3.6 mm and the side cut angle of the incision was set to 70° for all cases.

The carved lenticules had optical zones ranging from 5.5 to 6.0 mm based on the residual stromal depth which was always kept above 300 *μ*m. Standardized lenticule data for all cases included a transition zone of 0.1 mm, a minimum lenticule edge thickness of 10 *μ*m, and a circumferential side cut angle of 130°.

During surgery, the laser suction cup was centered relative to the pupil and the graft. The patient was asked to keep looking at the flickering green fixation light during laser application to the cornea. After creation of the cuts, the lenticule and the cap were manipulated using the usual techniques described for SMILE [34, 36].

After extraction, the lenticule was spread on the corneal surface and stained with prednisolone acetate 1% eye drops to ensure its intactness as a complete disc and to detect the presence of any residual tissue remnants within the intrastromal pocket that can result in irregular astigmatism [37].

The same postoperative treatment regimen consisting of topical prednisolone acetate 1%, gatifloxacin 0.3%, and nonpreserved artificial tears was followed for all patients.

### 2.4. Statistical Analysis

Data analysis was performed using the software SPSS for Windows version 20.0 (SPSS Inc., Chicago, USA) and Microsoft Excel 2010 (Microsoft corp., Redmond, WA, USA). Vector analysis for astigmatism results was done using Dr. Peyman's astigmatic vector analyzer software (http://www.drpeyman.ir/Ophthalmology_Calculator.htm). Nonparametric tests were used as the sample size was less than that optimum for parametric analysis. The Wilcoxon signed ranks test was used for comparison between the preoperative and postoperative data, and Kruskal-Wallis test was used for comparison between the postoperative data obtained from consecutive visits. Differences were considered to be statistically significant when the associated *p* value was <0.05. Bivariate regression analysis was carried out to predict achieved sphere, cylinder, and SEQ accuracy using the preoperative attempted data. Spearman correlation coefficient was used to assess the correlation between different variables. Standard graphs for reporting the outcomes in refractive surgery according to the Waring protocol and its modifications [38–40] were used for displaying and summarizing the outcomes of this study. For simplicity, only the preoperative and the 6-month follow-up data are demonstrated in the results.

## 3. Results

Demographic and pre- and postoperative clinical (refractive and visual) data of the ten patients are shown in Table 1. The mean age of the patients was 29 ± 3.4 years. The study included six females and 4 males.

In the majority of cases, the cap and the lenticule were centered in relation to the pupil within the corneal graft except in two cases (*n* = 2, 5) in whom the graft itself was slightly decentered. Here the best centration in relation to the pupil was performed taking into consideration not to bisect the graft-host interface with the laser incisions. It is, however, worth mentioning that those two cases were the ones in whom astigmatic correction was unlikely, with almost the same amount of astigmatism remaining postoperatively.

Intraoperatively, some resistance was met during dissection of both the cap and the lenticule at the sites of the suture related fibrous tracks. However, this has not led to any complication and cases were completed as planned.

None of the cases enrolled in the study suffered from any of the reported post-SMILE complications.

### 3.1. Refractive Outcomes

The preoperative mean spherical error was −5.3 ± 1.14 D (ranging from −7.0 to −4.0 D) and the preoperative mean refractive spherical equivalent (MRSE) was −6.84 ± 1.38 D (ranging from −9.0 to −5.0). On the other hand, the mean postoperative spherical refraction was −0.8 ± 0.97 D (ranging from −2.0 to +1.0 D) while the mean postoperative MRSE was −1.39 ± 0.9 D (ranging from −2.63 to 0.0 D).

The means of achieved correction values (calculated by subtracting the 6-month postoperative refraction from the preoperative target refraction) for sphere and MRSE were −4.5 ± 1.45 D and −5.45 ± 1.59 D, respectively. On comparing these values to the values of the preoperative target refractive correction (preoperative manifest refraction), statistically significant differences existed for both sphere (*p* = 0.042) and MRSE (*p* = 0.008).

Vector analysis of the results of astigmatism correction revealed a mean target induced astigmatism (TIA) magnitude of 2.61 ± 1.06 D at axis 66 ± 49.1 degrees and a mean surgically induced astigmatism (SIA) magnitude of 2.06 ± 0.76 D at axis 91.6 ± 59.3 degrees, while the mean of the magnitude of the difference vector was 1.14 ± 0.75 at axis 72.5 ± 53.8 degrees. On comparing the magnitudes of TIA versus SIA, a statistically significant difference was found (*p* = 0.028).

The mean correction ratio (induced/intended correction) of sphere was 0.84 ± 0.19 D and MRSE was 0.79 ± 0.13 D. This means that for each diopter of sphere about 84.3% correction was achieved and for each diopter in MRSE 79% correction was achieved. On the other hand, vector analysis of astigmatic results showed a mean percentage of astigmatic correction of 80.66 ± 20.9% (correction index = 0.81 ± 0.21) and a mean percentage of astigmatism reduction at the intended axis of 67 ± 25.5%. The mean arithmetic angle of error was −10.4 ± 15.4 degrees (*p* = 0.05) and the mean absolute angle of error was 12.4 ± 13.7 degrees (*p* = 0.008). The overall mean percentage of success of astigmatism surgery was 53 ± 37.9%.

**Figure 1:**
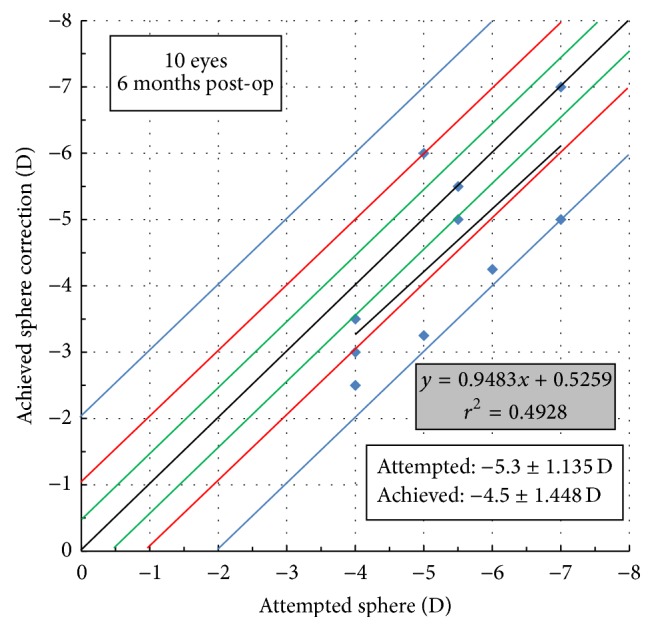
Attempted versus achieved spherical correction 6 months postoperatively.

**Figure 2:**
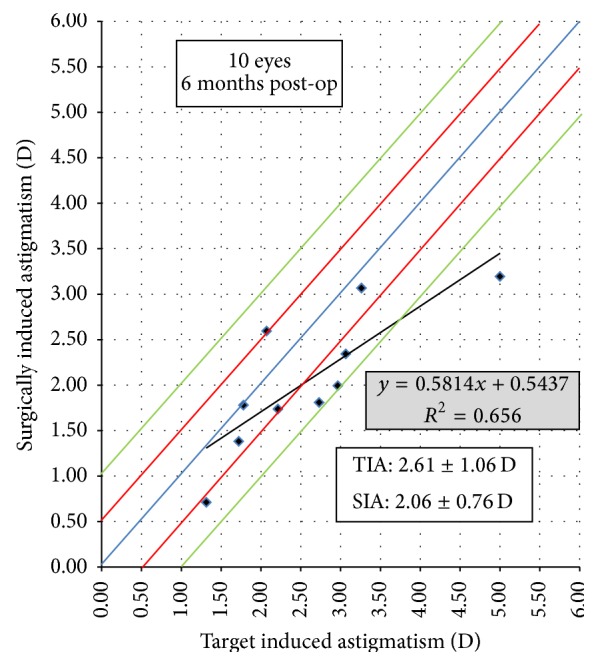
Target induced astigmatism versus surgically induced astigmatism 6 m postoperatively.

**Figure 3:**
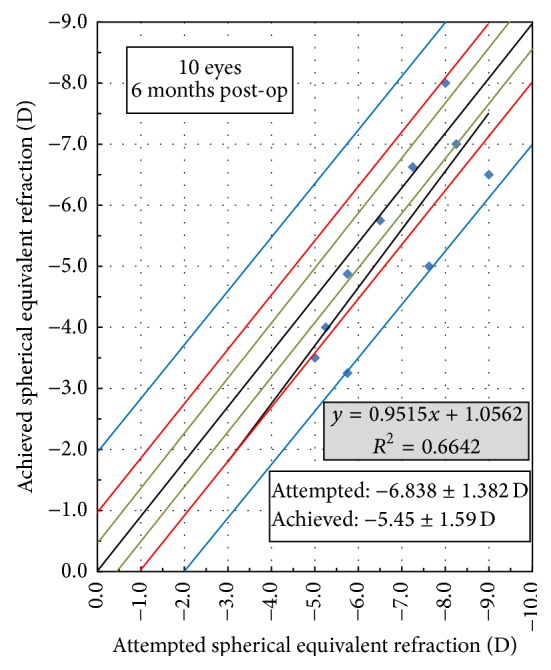
Attempted versus achieved spherical equivalent 6 m postoperatively.

Assessment of the accuracy of the achieved correction values versus the attempted ones revealed a positive correlation for all three refraction elements with the sphere showing correlation values of *p* = 0.024, *r* = 0.702, and *r* ^2^ = 0.4928 (Figure 1), the cylinder (TIA versus SIA) of *p* = 0.002, *r* = 0.855, and *r* ^2^ = 0.656 (Figure 2), and the MRSE of *p* = 0.004, *r* = 0.815, and *r* ^2^ = 0.6642 (Figure 3).

**Figure 4:**
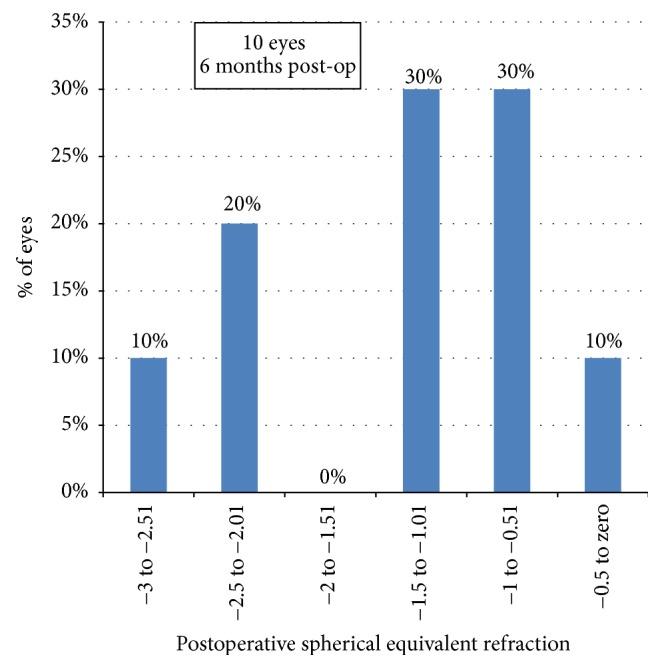
Predictability of spherical equivalent correction 6 m postoperatively.

As regards predictability, only one out of the ten eyes (10%) had a MRSE between zero and −0.5 D, 4 eyes (40%) were between zero and −1.0 D, and 7 eyes (70%) were between zero and −1.5 (Figure 4).

**Figure 5:**
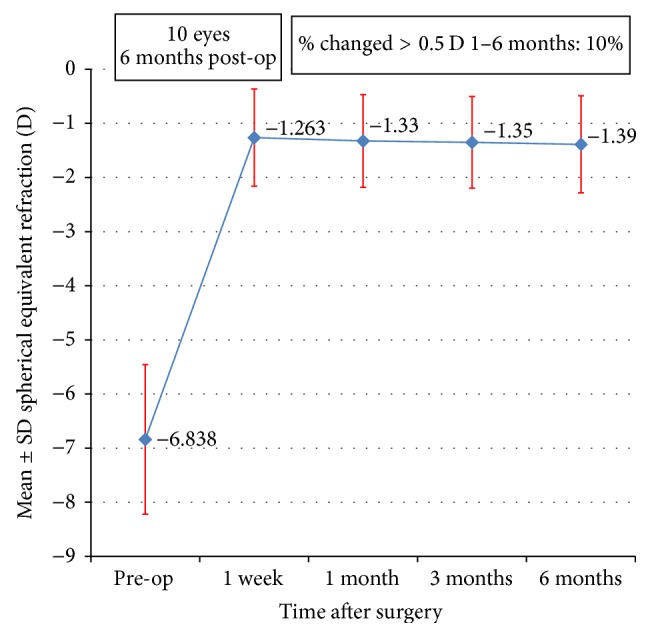
Stability of spherical equivalent refraction 6 m postoperatively.

Figure 5 displays the stability of the MRSE values along the follow-up period with a fairly stable refraction. No statistically significant differences were found among the MRSEs measured at one week (−1.26 ± 0.9), one month (−1.33 ± 0.86), three months (−1.35 ± 0.85), and six months (−1.39 ± 0.9) (*p* = 0.937).

**Figure 6:**
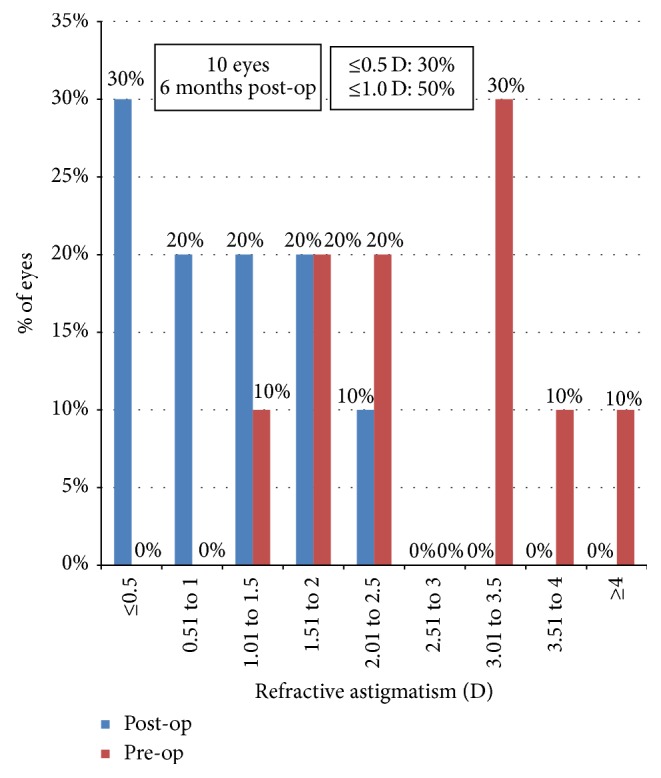
Predictability of refractive astigmatism correction 6 m postoperatively.

On assessing the predictability of astigmatism correction, five out of the ten eyes (50%) had astigmatism above 3.0 D preoperatively, while six months after surgery 30% had astigmatism values within 0.5 D, 50% within 1.0 D, and 70% within 1.5 D (Figure 6).

### 3.2. Visual Outcomes

Preoperatively, the mean CDVA was 0.73 ± 0.15 while, 6 months postoperatively, the mean UDVA was 0.68 ± 0.14 and the mean CDVA was 0.82 ± 0.1. This resulted in an efficacy index (**e**) of 0.93 and a safety index (**s**) of 1.12.

**Figure 7:**
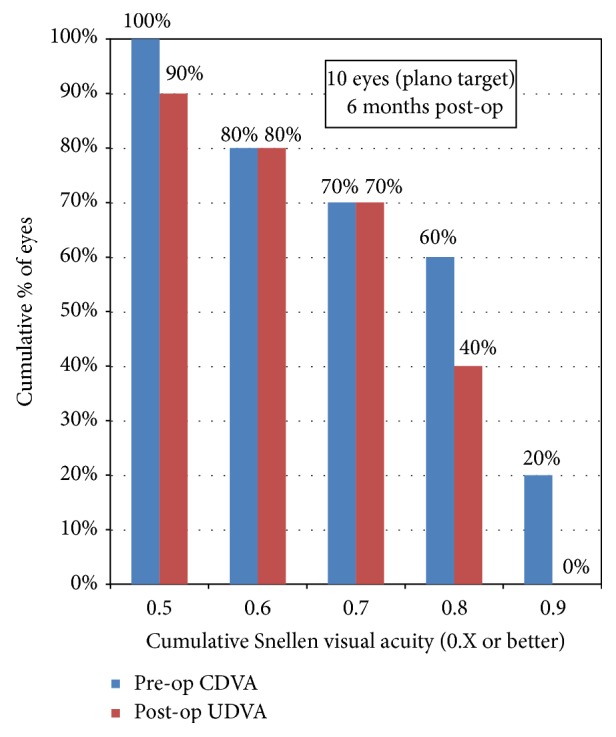
Uncorrected distance visual acuity 6 m postoperatively.

A more detailed evaluation of efficacy showed a cumulative Snellen preoperative CDVA of 0.9 or better in 20% of eyes, 0.8 or better in 60% of eyes, and 0.7 or better in 70% of eyes. On the other hand, the cumulative Snellen postoperative UDVA was 0.8 or better in 40% of eyes and 0.7 or better in 70% of eyes (Figure 7). Also, 90% of eyes had an UDVA within one line of the preoperative CDVA.

**Figure 8:**
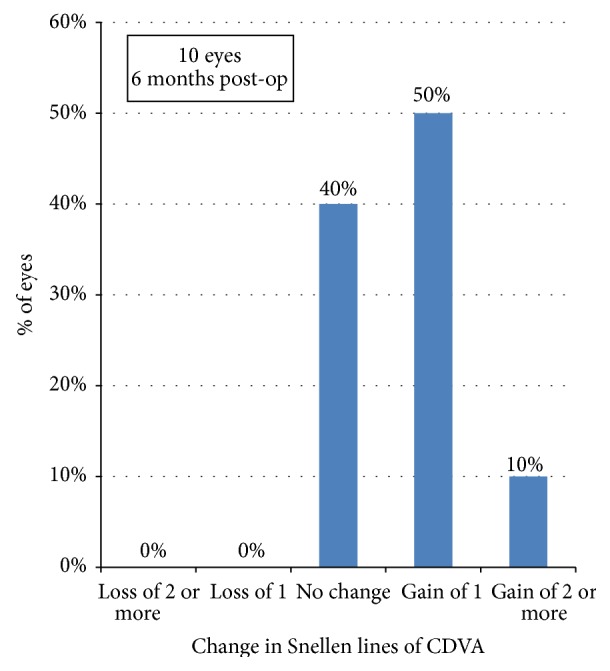
Change in corrected distance visual acuity 6 m postoperatively (safety).

Figure 8 shows the safety data of the procedure with 40% of eyes experiencing no change from the preoperative CDVA, 50% gaining one line, and 10% (one eye) gaining more than 2 lines. None of the enrolled eyes lost lines from their preoperative CDVA.

## 4. Discussion

The quest for the best unaided visual performance following PKP has entailed the exploration of a variety of conventional and novel refractive correction procedures. Yet, none has proven enough refractive accuracy, predictability, efficacy, or safety to be adopted as the standard trustworthy technique. A wide variability as regards the obtained refractive and visual results in post-PKP eyes compared to results reported in healthy eyes with unoperated corneas has become a generally anticipated conclusion for all reports on such cases. In addition, the inherent complications of these refractive surgical correction techniques were found to have a higher incidence rate in grafted corneas adding an increased menace for the future viability of the graft and, rarely, the whole eye [1–32].

The introduction of the single step femtosecond laser small incision lenticule extraction (SMILE) for correction of myopia and astigmatism and its reported good results gave hope for a simple, fast, easily designed, readily centered, and theoretically safe refractive correction means that can be applied to corneal grafts while salvaging the circumferential graft-host interface scar as well as the endothelium from being violated [33–35, 41–43].

This study was performed with the aim of evaluating visual and refractive outcomes after correcting postkeratoplasty myopia and myopic astigmatism using small incision lenticule extraction (SMILE).

To our knowledge, the only published data about SMILE after keratoplasty is a single case report in which the authors reported achieving the target refraction and an improved UDVA with a follow-up of 3 months. This study should, therefore, be one of the earliest clinical trials about the same subject [44].

The timing of intervention has been a matter of debate among researchers; however, it is generally agreed upon that the corneal graft-host junction heals completely about one year following transplantation and that further surgical interventions should not be done until three to four months has passed since all the sutures have been removed. Our cases had a minimum of 18 months before complete suture removal and refractive stabilization were pursued for three months afterwards [45].

As regards the refractive results, a statistically significant undercorrection was noted for sphere, cylinder, and MRSE. SMILE, in otherwise healthy nonoperated eyes, was reported to result in a slight undercorrection by about 0.25 D of MRSE as reported by Hjortdal et al. [36]. Possible causes suggested to explain such an undercorrection after SMILE for treating myopia included a small difference in the achieved lenticule thickness of about 9 *μ*m (due to a hypothesized elastic recoil of the lamella between the cap and the residual stromal bed) and postoperative epithelial thickness changes [46]. SMILE undercorrection of astigmatism was also reported by Ivarsen and Hjortdal in unoperated eyes especially for higher degrees of astigmatism (up to 16% per diopter in highly astigmatic eyes) [47]. Proposed mechanisms included lenticule decentration, inappropriate energy and spot spacing settings, and again postoperative epithelial hyperplasia. In addition to the previous causes, corneal button decentration as well as the release of the tension within the graft caused by dissecting the planar incisions and the sutures-related fibrous tracts can also have a role in undercorrection or induction of lower and/or higher order astigmatism and coma. Therefore, centration of the treatment in relation to the graft and the pupil is of paramount importance for achieving the best possible astigmatic correction and reducing higher order coma induction [48]. However, with decentered grafts, this might not be possible.

The statistically significant values of arithmetic and absolute angles of error of astigmatism correction denote the possibility cyclotorsion occurrence which can, also, aid the explanation of the relative imprecision of astigmatism correction at the intended angle as well as the induction of postoperative different axes cylindrical errors. Means for prevention of, compensating for, or correction of intraoperative cyclotorsion should be adopted.

Compared to other techniques, undercorrection and, rarely, overcorrection have also been reported for almost all corneal refractive surgical methods of correcting post-PKP myopia and astigmatism including incisional surgeries [12], photoablation (PRK or LASIK) [19–26], and ICR implantation [17, 18].

Despite this undercorrection reported for SMILE, stability of the achieved refraction was statistically proven on comparing the achieved MRSEs at 1 week, 1 month, 3 months, and six months. The same was reported by other studies which investigated SMILE for myopia and myopic astigmatism [33–35, 41–43]. The early biomechanical stability status achieved after SMILE can aid the explanation of such a finding [49].

Our results also showed that SMILE for correcting post-PKP myopia and astigmatism is of high efficacy and safety. The value of the efficacy index (**e**: 0.93) suggests that grafted patients undergoing SMILE can expect an UDVA of more than 90% of their preoperative CDVA, while the value of the safety index (**s**: 1.12) indicates a potential improvement of the CDVA postoperatively for such patients. These achieved values are comparable to those reported by Lin et al. [43] (**e**: 1.04, **s**: 1.01) and Hjortdal et al. [36] (**e**: 0.9, **s**: 1.07) who assessed SMILE for correcting myopia in nonoperated eyes. The fact that none of the eyes enrolled in the study had lost any of its preoperative CDVA postoperatively adds more to the safety profile of the technique.

The feasibility of centering the whole treatment within the graft had the advantage of avoiding the violation of the graft-host interface, thus, preserving the structural integrity of this potentially weak spot. On the contrary, in other nonfemtosecond laser dependent techniques, flap creation can easily breach (and consequently weakens) the circumferential scar [1]. Moreover, the keratoplasty scar is recognized as a new limbus [1, 50] outside which any refractive correction should be almost worthless and unquestionably risky. Any extension of the treatment procedure into the recipient's possibly diseased corneal rim either through flap lifting or laser ablation is, therefore, considered undesirable and quite useless. Also, the facts that the endothelium is spared in femtosecond refractive lenticule extraction procedures compared to other intraocular procedures [51] with no extra-stress added on the endothelial cells to achieve proper flap adhesion as in LASIK add another advantage to SMILE when compared to those refractive correction techniques [21, 27–32].

The drawbacks of this study, however, include the absence of controls, the lack of randomization, the few number of the enrolled eyes, and multiple surgeons. Since higher order aberrations evaluation and visual quality assessment were beyond the scope of this study, we strongly advocate them to be done in future similar studies to ascertain the nature and the amount of induced higher order aberrations as well as the quality of vision provided following SMILE in grafted eyes. The need for more prolonged follow-up, evaluation of induced graft biomechanical changes, and comparison to other techniques used for the same indication cannot be overlooked.

To sum up, SMILE for correction of postkeratoplasty myopia and astigmatism can be considered a valuable addition to the armamentarium of procedures utilized to correct post-PKP myopia and astigmatism. It is effective, safe, and stable with moderate accuracy and predictability. The whole treatment can be centered within the graft preserving the viability of the healed graft-host interface. However, management of cyclotorsion as well as centration of both the graft initially and the lenticule afterwards is crucial for achieving the best refractive results.

## Figures and Tables

**Table 1:** Demographic, refractive, and visual data of postkeratoplasty smile patients.

Patient	Gender	Age	Manifest refraction						Visual acuity			
			Preoperative			6 months			Preoperative		6 months	
			Sphere	Cylinder	Axis	Sphere	Cylinder	Axis	UDVA	CDVA	UDVA	CDVA
1	M	28	−4	−3.5	110	−0.5	−0.75	115	0.2	0.8	0.7	0.9
2	M	31	−5	−1.5	25	−1.75	−1.5	40	0.1	0.8	0.7	0.8
3	F	27	−7	−4	60	−2	−1	25	0.05	0.6	0.6	0.7
4	F	25	−6	−3.25	10	−1.75	−1.75	30	0.1	0.5	0.4	0.6
5	M	33	−7	−2.5	35	0	−2.5	70	0.1	0.7	0.7	0.9
6	F	35	−5.5	−2	70	−0.5	−0.5	90	0.1	0.8	0.8	0.9
7	F	25	−4	−2.5	65	−1	−0.5	60	0.3	0.9	0.8	0.9
8	F	28	−4	−2	15	−1.5	0	0	0.2	0.8	0.8	0.8
9	M	27	−5	−6	105	1	−2	115	0.05	0.5	0.8	0.8
10	F	31	−5.5	−3.5	165	0	−1.25	180	0.1	0.9	0.5	0.9

M: male, F: female, UDVA: uncorrected distance visual acuity, and CDVA: corrected distance visual acuity.
